# Hyaluronic Acid-Coated Chitosan Nanoparticles as an Active Targeted Carrier of Alpha Mangostin for Breast Cancer Cells

**DOI:** 10.3390/polym15041025

**Published:** 2023-02-18

**Authors:** Lisna Meylina, Muchtaridi Muchtaridi, I Made Joni, Khaled M. Elamin, Nasrul Wathoni

**Affiliations:** 1 Department of Pharmaceutics and Pharmaceutical Technology, Faculty of Pharmacy, Universitas Padjadjaran, Bandung 45363, Indonesia; 2 Department of Pharmaceutics and Pharmaceutical Technology, Faculty of Pharmacy, Universitas Mulawarman, Samarinda 75119, Indonesia; 3 Department of Pharmaceutical Analysis and Medicinal Chemistry, Faculty of Pharmacy, Universitas Padjadjaran, Bandung 45363, Indonesia; 4 Department of Physics, Faculty of Mathematics and Natural Sciences, Universitas Padjadjaran, Bandung 45363, Indonesia; 5 Functional Nano Powder University Center of Excellence, Universitas Padjadjaran, Bandung 45363, Indonesia; 6 Graduate School of Pharmaceutical Sciences, Kumamoto University, 5-1 Oe-honmachi, Chuo-ku, Kumamoto 862-0973, Japan

**Keywords:** alpha mangostin, chitosan, hyaluronic acid, polymeric nanoparticle, cytotoxic

## Abstract

Alpha mangostin (AM) has potential anticancer properties for breast cancer. This study aims to assess the potential of chitosan nanoparticles coated with hyaluronic acid for the targeted delivery of AM (AM-CS/HA) against MCF-7 breast cancer cells. AM-CS/HA showed a spherical shape with an average diameter of 304 nm, a polydispersity index of 0.3, and a negative charge of 24.43 mV. High encapsulation efficiency (90%) and drug loading (8.5%) were achieved. AM released from AM-CS/HA at an acidic pH of 5.5 was higher than the physiological pH of 7.4 and showed sustained release. The cytotoxic effect of AM-CS/HA (IC_50_ 4.37 µg/mL) on MCF-7 was significantly higher than AM nanoparticles without HA coating (AM-CS) (IC_50_ 4.48 µg/mL) and AM (IC_50_ 5.27 µg/mL). These findings suggest that AM-CS/HA enhances AM cytotoxicity and has potential applications for breast cancer therapy.

## 1. Introduction

Breast cancer is the most common type of malignancy and the second leading cause of cancer-related death worldwide [1,2]. In general, the treatment of breast cancer involves various combinations of surgery, radiation therapy, chemotherapy, and hormone therapy that have many drawbacks, such as limited effectiveness and unwanted side effects. In addition, chemotherapy shows low efficacy due to multidrug resistance and is highly toxic to healthy cells due to its non-specific targeting [3,4,5].

Alpha mangostin is a derivative of xanthone compounds isolated from the rind of the mangosteen fruit (*Garcinia mangostan*). AM has antiproliferative activity and apoptotic effects on different types of cancer, one of which is breast cancer, among the mechanisms of inducing apoptosis in breast cancer cells through the downregulation of B-cell lymphoma 2 (Bcl2) and the upregulation of Bcl-2-associated X protein (Bax) against breast cancer cells [6,7,8,9,10]. In addition to its anticancer activity, AM has limitations due to its poor solubility [11], the first fast metabolism reaction, efflux reactions caused by intercellular transporters, rapid drug release, and low selectivity for cancer cells [6,12,13,14].

The advancement of nanoparticle delivery system technology has the potential to improve delivery efficiency while minimizing side effects by directly targeting cancer cells [15,16,17,18,19]. Polymeric nanoparticles are a drug delivery system approach that utilizes polymers as carriers in the form of nanoparticles [20,21,22]. Polymeric nanoparticles are frequently produced from biopolymers such as chitosan in their formulation because they offer several advantages over other synthetic polymers, such as being green economy-friendly, eco-friendly, easy to make, bio-compatible, biodegradable, and low in their toxicity [23,24]. They have also been investigated for their ability to increase drug macromolecular permeation on epithelial membranes through the reversible opening of the transmembrane gap (tight junction) [25,26]. Chitosan nanoparticles have previously been used as AM carriers for breast cancer cells. In this study, AM was successfully encapsulated in chitosan nanoparticles, significantly increasing the cytotoxicity of AM (IC_50_ 6.7 µg/mL) compared to that which was not prepared in the polymeric nanoparticle formulation (IC_50_ 8.2 µg/mL) against the MCF-7 cell line [27,28].

Modification of nanoparticles aims to improve drug targeting through passive or active targeting [29,30]. Passive targeting can enhance the penetration of nanoparticles to the tumor tissue site through an enhanced permeability and retention (EPR) effect [31,32,33,34,35]. Meanwhile, active targeting contains structural modifications and surface functionalization of nanoparticles that lead to more specific targeting capabilities [36,37]. The limited selectivity of nanoparticles against cancer excludes the benefits of nanoparticle drug delivery for effective chemotherapy. It is critical to improve the selectivity of nanoparticles for cancer cells so that they can deliver more therapeutic agents to targeted cells than healthy cells, boosting therapeutic efficacy and minimizing adverse effects [38]. Therapeutic targeting can be accomplished by decorating the surface of nanoparticles with specific ligands to target the appropriate receptor cells, which are overexpressed on cancer cell membranes [39,40]. There are many candidates for ligand targeting, such as folate, antibodies, and hyaluronic acid, which have shown efficacy in breast cancer targeting [41,42]. Several studies have found that hyaluronic acid (HA) is one of the most often utilized ligands for coating chitosan nanoparticles for targeting breast cancer. HA is a natural polysaccharide made up of D-glucuronic acid and *N*-acetyl-D-glucosamine, which shows a high affinity for the integral membrane glycoprotein cluster differentiation-44 (CD44) on the cell surface in breast cancer [43,44]. CD44 is a cell surface receptor that is overexpressed in breast cancer, and targeting this receptor could facilitate intracellular uptake of nanoparticles, thereby increasing drug concentrations in cancer cells through CD44 receptor-mediated endocytosis [45,46,47,48,49,50,51].

In this study, HA-coated AM nanoparticles were developed and applied to actively target MCF-7 breast cancer cells that express CD44. For this purpose, the cytotoxic effect of HA-coated AM nanoparticles will be compared with that of AM and AM nanoparticles without HA.

## 2. Materials and Methods

### 2.1. Material

AM was obtained from Chengdu Biopurify Phytochemicals (Chengdu, Sichuan, China). Chitosan (CS), with MW: 1526.5 g/mol and DD: 81.38%, was isolated with a purity of 70%. HA (MW = 60 KDa) was purchased from Kangcare Bioindustry (Nanjing, China), and sodium tripolyphosphate (TPP) from Kristata (Bandung, West Java, Indonesia). The MCF-7 breast cancer cell line was obtained from the American Type Culture Collection (Manassas, VA, USA).

### 2.2. Method

#### 2.2.1. Fabrication of AM-CS

The ionic gelation technique was used to produce AM-CS. Briefly, AM (1 mg/mL) was dissolved in ethanol, and CS (1 mg/mL) was dissolved in acetic acid, then stirred overnight at room temperature with a magnetic stirrer, respectively. TPP (1 mg/mL) was dissolved into demineralized water. AM and CS solutions were mixed and transferred drop-by-drop to TPP solutions while being constantly magnetically stirred. The mixture was kept on a magnetic stirrer overnight at room temperature, then sonicated for 30 min. Finally, nanoparticles were separated from the mixture by centrifugation at 13,552× *g* for 30 min [27,28].

#### 2.2.2. Fabrication of Surface Functionalization of AM-CS

For the coating process, AM-CS and HA were dispersed in an acetate buffer at pH 5. Then, AM-CS was added dropwise to various concentrations of HA (Table 1) with constant vigorous stirring (30 min, 1200 rpm). The nanoparticles were then purified by centrifugation at 13,552× *g* for 30 min [42,52,53].

#### 2.2.3. Particle Size, Polydispersity Index (PDI), and Zeta Potential

The particle size, PDI, and zeta potential of the AM nanoparticles’ formulation were evaluated using the dynamic light scattering (DLS) analyzer (SZ 100 Horiba, Kyoto, Japan) [54,55].

#### 2.2.4. Morphology Studies

The morphology of AM-CS and AM-CS/HA was examined by scanning electron microscopy (SEM) (Model SU3500 SEM; Hitachi, Tokyo, Japan). The samples were placed into the stub and coated with platinum (30 s, 10 mA). AM-CS and AM-CS/HA photomicrographs were taken at 10 kV with 20,000 magnifications [27,28].

#### 2.2.5. Determination of Entrapment Efficiency and Drug Loading

The entrapment efficiency (EE) and drug loading (DL) of nanoparticles were calculated by spectroscopy. Briefly, AM-CS/HA was mixed with ethyl acetate and centrifuged (6000× *g* rpm, 5 min). After collecting the supernatant, the absorbance at 245 nm was measured with a spectrophotometer. The supernatant was then resuspended in sufficient ethanol to determine the amount of AM encapsulated and the total amount of AM. Serial concentrations of AM (2–12 µg/mL) were measured at 245 nm to generate the standard curve. EE and DL of AM in AM-CS/HA were calculated by Equations (1) and (2) [27,28]:

(1) $$EE\;\left(\%\right)=\frac{mass\;of\;the\;AM\;in\;AM-CS/HA\;}{mass\;of\;AM\;used}\times 100\%\;$$

(2) $$DL\;\left(\%\right)=\frac{mass\;of\;the\;AM\;in\;AM-CS/HA\;\;}{mass\;of\;AM-CS/HA\;}\times 100\%$$

#### 2.2.6. Fourier-Transform Infrared Spectroscopy Analysis

The chemical interaction of raw materials and nanoparticles was investigated using a Fourier-transform infrared spectrophotometer (FTIR) (Thermo Fisher, Waltham, MA, USA) and measured at 4000–400 cm^−1^ [27,56].

#### 2.2.7. X-ray Diffraction Analysis

X-ray diffraction (XRD) (X-pert MPD diffractometer type, Rigaku International, Tokyo, Japan) was used to examine the crystallinity of AM-CS/HA. The samples were scanned throughout an angular range (2 theta) of 5–60° [27,57].

#### 2.2.8. Differential Scanning Calorimetry Analysis

Differential scanning calorimetry (DSC) (Perkin Elmer DSC-6, MA, USA) was used to study the thermal properties of AM-CS/HA. The samples were carried out at a heating rate of 10 °C/min from 30 to 300 °C, with a stream of flowing nitrogen at 50 mL/min [27].

#### 2.2.9. In Vitro Release Studies

The release profile in phosphate-buffered saline (PBS) solution was investigated at pH 7.4 and 5.5. Typically, 5 mg of nanoparticles were dispersed in PBS and transferred to a dialysis tube (molecular weight cut-off 12,000 Da). The dialysis tube was immersed in PBS medium before being put into a beaker containing 50 mL of release medium at 37 °C and 100 rpm. At determined time intervals, 5 mL of dissolution medium was taken and replaced with an equal quantity of fresh medium. The collected samples were then measured using a spectrophotometer at a wavelength of 245 nm [55].

#### 2.2.10. Cytotoxicity Studies

The MTT assay was used to assess the cytotoxic activity of AM and nanoparticles on MCF-7 cells. Here, 5000 cells/well of MCF-7 cells (ATCC) were seeded on 96-well plates in the presence of RPMI culture media containing 10% FCS for 24 h. Then, the media was aspirated and replaced with cell culture media containing various amounts of AM (2–6 μg/mL). Next, 0.5 mg/mL of MTT solution was added and incubated for 4 h at 37 °C. The formed formazan crystals were treated with 100 µL of SDS in 0.01% HCl, and then the absorbance was measured at 450 nm using an ELISA plate reader (EpochTM Microplate Spectrophotometer, VT, USA). Cell viability was represented as a percentage of the treated cells compared to the control cells, as stated in Equation (3), and IC_50_ was calculated from the dose–response curves [58]:

(3) $$Cell\;viability\;\left(\%\right)=\frac{absorbance\;of\;treated\;sample}{absorbance\;of\;control\;sample}\times 100\%$$

#### 2.2.11. Statistical Analysis

The quantitative data were expressed as the mean ± standard error of the mean (S.E.M.). The two-way ANOVA was used for statistical analysis. *p*-values < 0.05 were considered significant.

## 3. Results

### 3.1. Characterization of AM Nanoparticles

#### 3.1.1. Particle Size, PDI, Zeta Potential, Morphology, EE, and DL

The mean particle size, PDI, and zeta potential of various AM nanoparticle formulas are shown in Table 2. The data show that the nanoparticle size is in the range of 200–400 nm. The zeta potential of AM-CS showed a positive value, then the AM-CS/HA showed a negative value. In addition, the PDI of all formulas was <1. In this study, AM-CS/HA1 was selected for further characterization and cytotoxicity evaluation on MCF-7 cells because this formula produced the smallest particle size.

**Figure 1:**
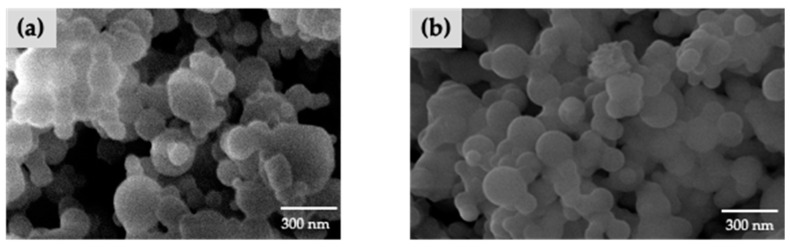
SEM photomicrographs of (**a**) AM-CS and (**b**) AM-CS/HA1.

The morphologies of nanoparticles were examined by SEM (Figure 1). As shown in Figure 1, the nanoparticles were approximately spherical. The EE and DL are displayed in Table 3. The average entrapment efficiency of the AM-CS and AM-CS/HA1 was 85.32% ± 0.40% and 90.40% ± 0.161%, respectively, indicating that AM did not escape from the nanoparticles during the HA coating process.

#### 3.1.2. FTIR Analysis

**Figure 2:**
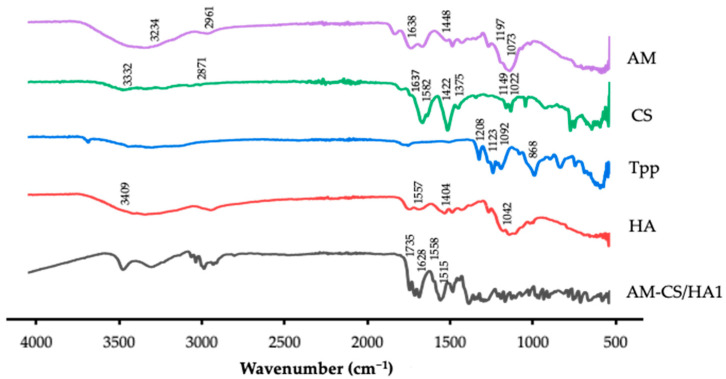
FTIR spectrum of raw material and AM-CS/HA1.

The results of the FTIR analysis are displayed in Figure 2. The AM spectrum showed the presence of O–H stretch at 3411.15 and 3234.88 cm^−1^, stretching vibrations of C–H at 2988.11, 2961.04, and 2910.40 cm^−1^, C=O at 1638.20 cm^−1^, C–C at 1448.33 cm^−1^, orto–OCH_3_ stretch at 1197.83 cm^−1^, and C–O–C stretch at 1073.82 cm^−1^ [59,60]. The CS spectrum displayed broad peaks around 3332.14 cm^−1^ corresponding to the amide (N-H) and O-H groups, C–H stretch at 2871.62 cm^−1^, C=O stretch at 1637.26 cm^−1^, N–H bend at 1582.86 cm^−1^, C–H bend at 1422.38 cm-1, C–N at 1375.93 cm^−1^, C–O–C stretch at 1149.98 cm^−1^, and C–O at 1022.44 cm^−1^ [61,62]. The characteristic absorption peaks of HA were 3409 cm^−1^ corresponding to the N–H and O–H groups, amide II and III at 1557 and 1337 cm^−1^, C–C stretching of the COONa group was observed at 1404 cm^−1^, and C–O stretch at 1042 cm^−1^ [63]. The spectra of AM-CS/HA1 presented absorption bands at 1515.21 and 1735.45 cm^−1^ due to –NH_3_ of CS and –COOH of HA, respectively [64].

#### 3.1.3. XRD Analysis

**Figure 3:**
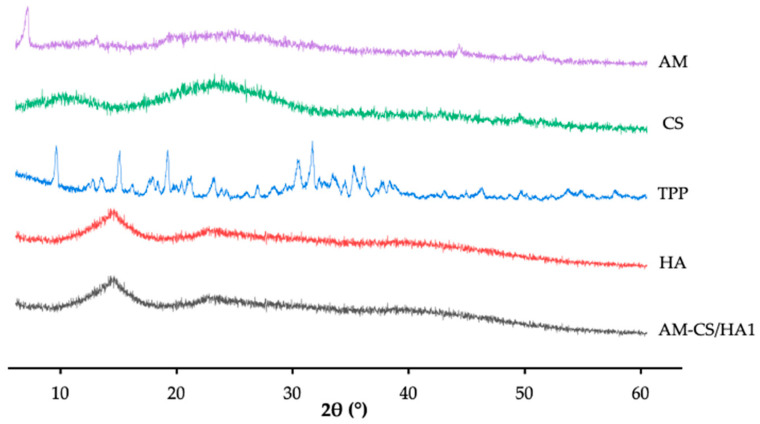
XRD patterns of raw material and AM-CS/HA1.

The XRD patterns are displayed in Figure 3. The AM showed sharp multiple peaks at 2θ of 5.4°, 11.6°, and 13.3°, which indicated a crystalline pattern [65,66], and the CS showed peaks at 10.4°, 19.7°, and 29.3° that exhibited semi-crystalline patterns [67,68,69]. The XRD spectrum of HA showed no specific diffraction pattern, indicating the amorphous nature of HA [64]. The peaks exhibited by the AM-CS/HA1 resembled those of HA and showed an amorphous nature.

#### 3.1.4. DSC Analysis

**Figure 4:**
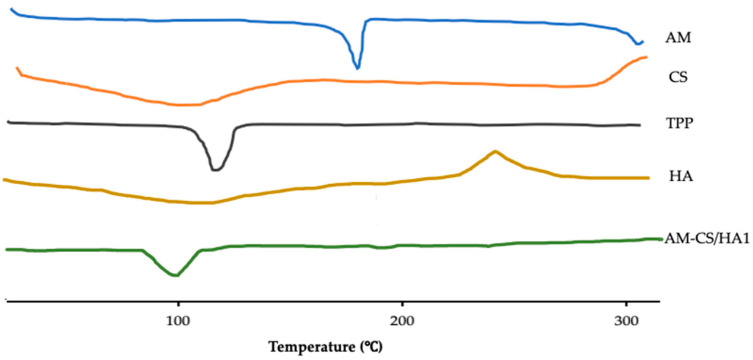
DSC thermographs of raw material and AM-CS/HA1.

DSC thermograms for AM exhibited the endothermic phase at 177 °C, and HA had an obvious glass transition peak at 85 °C and an exothermic peak at 241 °C. The DSC thermogram of chitosan showed an endothermic peak between 95.1 and 102.3 °C and an exothermic peak between 303.77 and 304.28 °C. The AM-CS/HA1 displayed patterns that corresponded to the glass transition (103.1 °C). The results of the DSC analysis are shown in Figure 4.

### 3.2. In Vitro Release Studies

**Figure 5:**
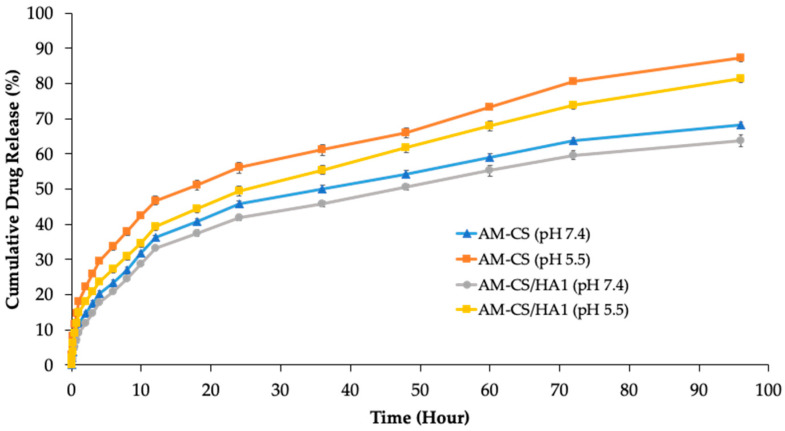
AM release profiles from AM-loaded nanoparticles at pH 7.4 and 5.5. Each value represents the mean ± S.E.M.

The profile of the in vitro release of AM from nanoparticles in PBS (pH 7.4 and 5.5) within 96 h is shown in Figure 5, and the Higuchi parameters for release kinetics are summarized in Table 4. The release of AM from nanoparticles demonstrated an initial burst of up to 11% during the first hour, followed by a sustained release for 96 h.

### 3.3. Cytotoxicity Studies

**Figure 6:**
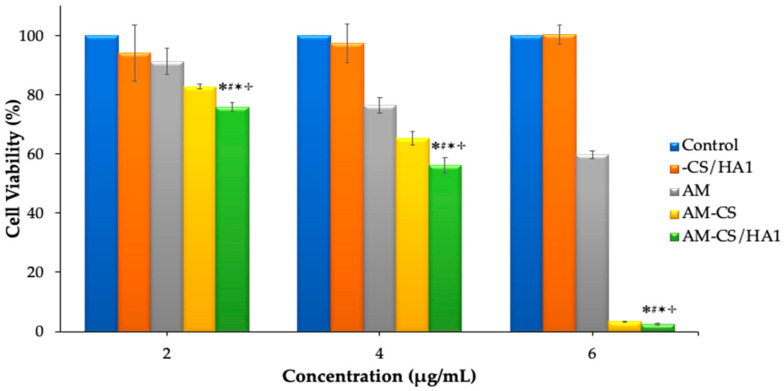
Cytotoxic activity of AM, -CS/HA1, AM-CS, and AM-CS/HA1 in MCF-7 cells. Each value represents the mean ± S.E.M. ✽ *p* < compared to the control; # *p* < compared to -CS/HA1; ✶ *p* < compared to AM; ✢ *p* < compared to AM-CS.

The cytotoxic activity of AM, -CS/HA1, AM, AM-CS, and AM-CS/HA1 was evaluated on MCF-7 cells, as shown in Figure 6. For -CS/HA, no cytotoxic activity was observed in MCF-7 cells. On the other hand, the cytotoxicity of AM, AM-CS, and AM-CS/HA significantly differed. AM, AM-CS, and AM-CS/HA had IC_50_ of 5.27, 4.48, and 4.37 μg/mL, respectively. Thus, these results demonstrated that AM-CS/HA has higher cytotoxicity compared to AM and AM-CS.

## 4. Discussion

AM has shown potentiality in the treatment of various types of cancer. Previous research has demonstrated that AM nanoparticles have a remarkable therapeutic impact on breast cancer [6,7]. In this study, AM-loaded nanoparticles were coated with HA for breast cancer targeting. Nanocarriers with tumor-targeting moiety attachments, such as hyaluronic acid, have the potential to increase tumor-targeted delivery, while minimizing pharmacological adverse effects [42,70,71,72,73].

In this study, AM-CS was fabricated with HA (AM-CS/HA) via an electrostatic deposition technique. As an experimental variable, three different concentrations of HA (20, 40, and 60 mg) were used. As shown in Table 2, there was a correlation between the variations in HA concentrations and particle size, or zeta potential. The higher concentration of HA resulted in larger particle sizes and a lower zeta potential value. At low concentrations, HA will enter more readily and deeply through the pores in AM-CS, resulting in denser particles, which will further increase the particle size when the HA concentration increases owing to the accumulation of the coated polymer chains on the exterior of the nanoparticles. The coating of AM nanoparticles resulted in a conversion of the nanoparticles’ surface charge. HA has been found to exert a negative charge on the nanoparticles’ surface because HA molecules are mostly located in the outermost shell of nanoparticles. Positively charged nanocarriers promote membrane attachment, uptake, and release of endosomes, while nanocarriers with a negative zeta potential exhibit more selective and efficient absorption, particularly when coated with targeting ligands [53,54,74].

The particle size of nanoparticles plays an important role in chemotherapeutic drug delivery systems because it can affect cellular uptake via endocytosis and determine their fate during systemic circulation [27]. Studies have shown that the nanoparticle size range between 40 and 400 nm is suitable for extending the circulation time and increasing drug accumulation in tumors [75]. This is a further reason for selecting AM-CS/HA1 for further investigation to evaluate its characteristics and cytotoxicity.

The spectrum of the AM-CS/HA1 exhibited some characteristic vibrations of HA and CS, then shifted to a higher wave number. The signal shift demonstrated that both macromolecular chains were involved in the production of the nanoparticles. The absorption band at 1735.45 cm^−1^ showed the protonation that occurred in the formation of the polyelectrolyte complex [63,76]. Moreover, the amplification of the peak corresponding to the amide I and II bands, with a small shift to wave numbers 1628.39 and 1558.62 cm^−1^, showed effective amide bonding between the amino and carboxylic groups on the HA and the surface of the nanoparticles [42,63,77].

The AM-CS/HA1 diffractogram data demonstrated the transformation of the crystalline or semi-crystalline phase of the material component into an amorphous form. The termination of the amine and hydroxy groups is thought to be the origin of CS’s semi-crystalline transition, resulting in the development of an amorphous complex with the coated polymer (HA). Furthermore, the AM crystal lattice no longer appeared, suggesting that AM has been uniformly dispersed and encapsulated in the system [27,78].

The DSC thermogram of AM nanoparticles coated with HA exhibited a loss of the peak from CS accompanied by shifting of the HA peak to 94 °C and a loss of the exothermic peak at 236 °C from HA, which is thought to be due to the structural modification of HA after electrostatic interaction with CS [77,79]. Furthermore, AM exhibited a significant endothermal peak around 178 °C due to the melting of AM crystals. However, the AM-CS/HA diffractogram did not show an endothermic peak of AM. It can be explained that the crystallization of AM is inhibited by the nanoparticle matrix, and AM may be in a molecular or amorphous state in the nanoparticle system [78].

The release profile of AM from the nanoparticle system exhibited biphasic behavior, with early and fast release phases, followed by sustained release. Both coated and uncoated nanoparticles showed an initial burst of AM release, which was related to the quick diffusion of free drug adsorbed on the particles [42,76]. The delayed release rate of HA-coated nanoparticles compared to uncoated nanoparticles indicated that the HA coating on the surface of the nanoparticles inhibits the diffusion of drugs trapped in the nanoparticle system to be released. It happens because the coating of HA on the surface of the nanoparticles increases their density and structural hardness due to increased cross-linking interactions between the constituent components, and reduces the release of the active substance [80]. Furthermore, because chitosan and hyaluronic acid are pH-sensitive polymers, pH influences the release of AM from nanoparticles [81]. Due to the breakdown in the electrostatic balance between CS and TPP in AM-CS or between CS, TPP, and HA in -CS/HA in an acidic environment, AM release was larger at pH 5.5 than at pH 7.4. This pH-dependent release mechanism reduces the drug’s systemic toxicity due to decreased bioavailability in healthy organs at physiological pH, which could reduce drug side effects for patients [82,83]. Subsequently, pH-sensitive drug delivery systems result in higher bioavailability for drugs at tumor sites at acidic pH and increase their efficiency in malignant tissues [54,76,84,85]. To estimate the kinetic profiles of AM release from the nanoparticle system, we carried out an analysis using the Higuchi model. Based on the value of the correlation coefficient (*r*), this indicated that the type of release of AM from nanoparticles was a matrix type, based on Fickian diffusion [80,86]. It is known that Higuchi’s kinetic model involves drug release from the polymer matrix system, which releases drugs in a controlled and sustainable manner [87,88]. This is very important for the release of chemotherapy drugs to reduce their toxicity [89,90].

The cytotoxic study on drug-free nanoparticles (-CS/HA1) showed activity on cell viability > 90% at all tested concentrations. These results indicate that the nanoparticle carrier exhibited good biocompatibility and was less toxic to the tested MCF-7 cells. In contrast, cells that were treated with AM, AM-CS, or AM-CS/HA1 demonstrated a dose-dependent response to the drug. Moreover, the cells utilized were more sensitive to AM-CS and HA than to AM and AM-CS. The cytotoxic activity of AM and AM-CS/HA1 at the same doses was significantly different (*p* < 0.05). In conclusion, AM has lower cytotoxicity than AM-CS/HA because the HA coating of nanoparticles interacts with the CD44 receptor and is then internalized via receptor-mediated endocytosis.

## 5. Conclusions

The development of targeted drug delivery systems is necessary for the delivery of anticancer drugs to reduce systemic side effects and increase the effectiveness of therapy. Surface-modified nanoparticle delivery systems using specific ligands, such as hyaluronic acid, to target cell receptors that are overexpressed on breast cancer cell membranes, such as the CD44 receptor, have the potential to increase the efficiency of anticancer drug delivery to breast cancer cells [42]. This research succeeded in developing a targeted delivery system of hyaluronic acid-coated chitosan nanoparticles for the targeted delivery of alpha mangostin for breast cancer. Our findings showed that alpha mangostin loaded in our delivery system had a significant impact on MCF-7 cancer cells at a lower dose (IC_50_ 4.37 μg/mL) compared to free alpha mangostin (IC_50_ 5.27 μg/mL) or nanoparticles of alpha mangostin with chitosan carriers without a hyaluronic acid coating (IC_50_ 4.48 μg/mL, IC_50_ 6.7 μg/mL [27], IC_50_ 4.90 μg/mL [91]). The most conclusive findings of this study indicated that the developed alpha mangostin targeted nanoparticle delivery system can be used as an effective treatment for breast cancer by specifically targeting cancer cells. Further research needs to be conducted in vivo to determine the bioavailability, toxicity, and anticancer activity of alpha mangostin nanoparticles coated with hyaluronic acid.

## Figures and Tables

**Table 1:** AM nanoparticles’ formulation.

Formulation	AM (mg/mL)	CS (mg/mL)	TPP (mg/mL)	HA (mg/mL)
AM-CS	1	10	2	-
AM-CS/HA1	1	10	2	20
AM-CS/HA2	1	10	2	40
AM-CS/HA3	1	10	2	60

**Table 2:** Particle size, distribution, and zeta potential of AM nanoparticles.

Formulation	Particle Size (nm)	PDI	Zeta Potential (mV)
AM-CS	229.133 ± 5.685	0.382 ± 0.015	33.83 ± 1.92
AM-CS/HA1	304.833 ± 6.288	0.362 ± 0.038	−24.43 ± 1.76
AM-CS/HA2	369.300 ± 2.467	0.360 ± 0.028	−28.44 ± 2.26
AM-CS/HA3	412.767 ± 6.001	0.346 ± 0.034	−33.31 ± 1.85

**Table 3:** The average of EE and DL of the nanoparticles.

Formulation	EE (%)	DL (%)
AM-CS	88.325 ± 3.340	8.674 ± 0.018
AM-CS/HA1	90.404 ± 2.161	8.514 ± 0.007

**Table 4:** Higuchi regression parameter for AM release from AM-CS and AM-CS/HA1.

Parameter	AM-CS		AM-CS/HA1	
	pH 7.4	pH 5.5	pH 7.4	pH 5.5
Intercept	0.852 ± 0.477	3.265 ± 0.129	0.889 ± 0.169	1.723 ± 0.3428
Slope	9.281 ± 0.489	11.821 ± 0.377	9.097 ± 0.160	10.289 ± 0.340
Correlation coefficient (*r*)	0.994 ± 0.002	0.981 ± 0.005	0.995 ± 0.001	0.987 ± 0.002

## Data Availability

The data presented in this research are accessible upon request from the corresponding author.

## References

[1] Giaquinto A.N., Sung H., Miller K.D., Kramer J.L., Newman L.A., Minihan A., Jemal A., Siegel R.L. (2022). Breast Cancer Statistics, 2022. CA Cancer J. Clin..

[2] Siegel R.L., Miller K.D., Fuchs H.E., Jemal A. (2022). Cancer Statistics, 2022. CA Cancer J. Clin..

[3] Hong R., Xu B. (2022). Breast Cancer: An up-to-Date Review and Future Perspectives. Cancer Commun..

[4] Luo Z., Dai Y., Gao H. (2019). Development and Application of Hyaluronic Acid in Tumor Targeting Drug Delivery. Acta Pharm. Sin. B.

[5] Feng Y., Spezia M., Huang S., Yuan C., Zeng Z., Zhang L., Ji X., Liu W., Huang B., Luo W. (2018). Breast Cancer Development and Progression: Risk Factors, Cancer Stem Cells, Signaling Pathways, Genomics, and Molecular Pathogenesis. Genes Dis..

[6] Kritsanawong S., Innajak S., Imoto M., Watanapokasin R. (2016). Antiproliferative and Apoptosis Induction of α-Mangostin in T47D Breast Cancer Cells. Int. J. Oncol..

[7] Shibata M.A., Iinuma M., Morimoto J., Kurose H., Akamatsu K., Okuno Y., Akao Y., Otsuki Y. (2011). α-Mangostin Extracted from the Pericarp of the Mangosteen (*Garcinia mangostana* Linn) Reduces Tumor Growth and Lymph Node Metastasis in an Immunocompetent Xenograft Model of Metastatic Mammary Cancer Carrying a P53 Mutation. BMC Med..

[8] Benjakul R., Kongkaneramit L., Sarisuta N., Moongkarndi P., Müller-Goymann C.C. (2015). Cytotoxic Effect and Mechanism Inducing Cell Death of α-Mangostin Liposomes in Various Human Carcinoma and Normal Cells. Anti-Cancer Drugs.

[9] Muchtaridi M., Wijaya C.A. (2017). Anticancer Potential of α-Mangostin. Asian J. Pharm. Clin. Res..

[10] Ibrahim M.Y., Hashim N.M., Mohan S., Kamalidehghan B., Ghaderian M., Dehghan F., Ali L.Z., Arbab I.A., Yahayu M., Lian G.E.C. (2014). α-Mangostin from Cratoxylum Arborescens Demonstrates Apoptogenesis in MCF-7 with Regulation of NF-ΚB and Hsp70 Protein Modulation in Vitro, and Tumor Reduction in Vivo. Drug Des. Devel. Ther..

[11] Meylina L., Muchtaridi M., Joni I.M., Mohammed A.F.A., Wathoni N. (2021). Nanoformulations of α-Mangostin for Cancer Drug Delivery System. Pharmaceutics.

[12] Verma R.K., Yu W., Shrivastava A., Shankar S., Srivastava R.K. (2016). α-Mangostin-Encapsulated PLGA Nanoparticles Inhibit Pancreatic Carcinogenesis by Targeting Cancer Stem Cells in Human, and Transgenic (KrasG12D, and KrasG12D/Tp53R270H) Mice. Sci. Rep..

[13] Gutierrez-Orozco F., Failla M.L. (2013). Biological Activities and Bioavailability of Mangosteen Xanthones: A Critical Review of the Current Evidence. Nutrients.

[14] Li L., Brunner I., Han A.R., Hamburger M., Kinghorn A.D., Frye R., Butterweck V. (2011). Pharmacokinetics of α-Mangostin in Rats after Intravenous and Oral Application. Mol. Nutr. Food Res..

[15] Aydin R.S.T. (2013). Herceptin-Decorated Salinomycin-Loaded Nanoparticles for Breast Tumor Targeting. J. Biomed Mater. Res.—Part A.

[16] Masood F. (2016). Polymeric Nanoparticles for Targeted Drug Delivery System for Cancer Therapy. Mater. Sci. Eng. C.

[17] Aghebati-Maleki A., Dolati S., Ahmadi M., Baghbanzhadeh A., Asadi M., Fotouhi A., Yousefi M., Aghebati-Maleki L. (2020). Nanoparticles and Cancer Therapy: Perspectives for Application of Nanoparticles in the Treatment of Cancers. J. Cell. Physiol..

[18] Ashfaq U.A., Riaz M., Yasmeen E., Yousaf M. (2017). Recent Advances in Nanoparticle-Based Targeted Drug-Delivery Systems against Cancer and Role of Tumor Microenvironment. Crit. Rev. Ther. Drug Carr. Syst..

[19] Pérez-Herrero E., Fernández-Medarde A. (2015). Advanced Targeted Therapies in Cancer: Drug Nanocarriers, the Future of Chemotherapy. Eur. J. Pharm. Biopharm..

[20] Madej M., Kurowska N., Strzalka-Mrozik B. (2022). Polymeric Nanoparticles—Tools in a Drug Delivery System in Selected Cancer Therapies. Appl. Sci..

[21] Perumal S. (2022). Polymer Nanoparticles: Synthesis and Applications. Polymers.

[22] Xiao X., Teng F., Shi C., Chen J., Wu S., Wang B., Meng X., Essiet Imeh A., Li W. (2022). Polymeric Nanoparticles—Promising Carriers for Cancer Therapy. Front. Bioeng. Biotechnol..

[23] Khan M.U.A., Razak S.I.A., Haider S., Mannan H.A., Hussain J., Hasan A. (2022). Sodium Alginate-f-GO Composite Hydrogels for Tissue Regeneration and Antitumor Applications. Int. J. Biol. Macromol..

[24] Khan M.U.A., Al-Arjan W.S., Ashammakhi N., Haider S., Amin R., Hasan A. (2022). Multifunctional Bioactive Scaffolds from ARX-g-(Zn@rGO)-HAp for Bone Tissue Engineering: In Vitro Antibacterial, Antitumor, and Biocompatibility Evaluations. ACS Appl. Bio Mater..

[25] Hassani S., Laouini A., Fessi H., Charcosset C. (2015). Preparation of Chitosan-TPP Nanoparticles Using Microengineered Membranes—Effect of Parameters and Encapsulation of Tacrine. Colloids Surfaces A Physicochem. Eng. Asp..

[26] Palanikumar L., Al-Hosani S., Kalmouni M., Nguyen V.P., Ali L., Pasricha R., Barrera F.N., Magzoub M. (2020). PH-Responsive High Stability Polymeric Nanoparticles for Targeted Delivery of Anticancer Therapeutics. Commun. Biol..

[27] Wathoni N., Meylina L., Rusdin A., Abdelwahab Mohammed A.F., Tirtamie D., Herdiana Y., Motoyama K., Panatarani C., Joni I.M., Lesmana R. (2021). The Potential Cytotoxic Activity Enhancement of α-Mangostin in Chitosan-Kappa Carrageenan-Loaded Nanoparticle against Mcf-7 Cell Line. Polymers.

[28] Wathoni N., Rusdin A., Febriani E., Purnama D., Daulay W., Azhary S.Y., Panatarani C., Joni I.M., Lesmana R., Keiichi M. (2019). Formulation and Characterization of α-Mangostin in Chitosan Nanoparticles Coated by Sodium Alginate, Sodium Silicate, and Polyethylene Glycol. J. Pharm. Bioallied Sci..

[29] Alavi M., Hamidi M. (2019). Passive and Active Targeting in Cancer Therapy by Liposomes and Lipid Nanoparticles. Drug Metab. Pers. Ther..

[30] Tu Y., Yao Z., Yang W., Tao S., Li B., Wang Y., Su Z., Li S. (2022). Application of Nanoparticles in Tumour Targeted Drug Delivery and Vaccine. Front. Nanotechnol..

[31] Kher C., Kumar S. (2022). The Application of Nanotechnology and Nanomaterials in Cancer Diagnosis and Treatment: A Review. Cureus.

[32] Choi K.A., Kim J.H., Ryu K., Kaushik N. (2022). Current Nanomedicine for Targeted Vascular Disease Treatment: Trends and Perspectives. Int. J. Mol. Sci..

[33] Yang T., Zhai J., Hu D., Yang R., Wang G., Li Y., Liang G. (2022). “Targeting Design” of Nanoparticles in Tumor Therapy. Pharmaceutics.

[34] Sun Y., Yang Q., Xia X., Li X., Ruan W., Zheng M., Zou Y., Shi B. (2021). Polymeric Nanoparticles for Mitochondria Targeting Mediated Robust Cancer Therapy. Front. Bioeng. Biotechnol..

[35] Drozdov A.S., Nikitin P.I., Rozenberg J.M. (2021). Systematic Review of Cancer Targeting by Nanoparticles Revealed a Global Association between Accumulation in Tumors and Spleen. Int. J. Mol. Sci..

[36] Peltonen L., Singhal M., Hirvonen J. (2020). Principles of Nanosized Drug Delivery Systems.

[37] Dadwal A., Baldi A., Kumar Narang R. (2018). Nanoparticles as Carriers for Drug Delivery in Cancer. Artif. Cells Nanomed. Biotechnol..

[38] Zhu R., Zhang F., Peng Y., Xie T., Wang Y., Lan Y. (2022). Current Progress in Cancer Treatment Using Nanomaterials. Front. Oncol..

[39] Zhou Z., Badkas A., Stevenson M., Lee J.Y., Leung Y.K. (2015). Herceptin Conjugated PLGA-PHis-PEG PH Sensitive Nanoparticles for Targeted and Controlled Drug Delivery. Int. J. Pharm..

[40] Jurj A., Braicu C., Pop L.A., Tomuleasa C., Gherman C.D., Berindan-Neagoe I. (2017). The New Era of Nanotechnology, an Alternative to Change Cancer Treatment. Drug Des. Devel. Ther..

[41] Dosio F., Arpicco S., Stella B., Fattal E. (2016). Hyaluronic Acid for Anticancer Drug and Nucleic Acid Delivery. Adv. Drug Deliv. Rev..

[42] Hashad R.A., Ishak R.A.H., Geneidi A.S., Mansour S. (2017). Surface Functionalization of Methotrexate-Loaded Chitosan Nanoparticles with Hyaluronic Acid/Human Serum Albumin: Comparative Characterization and in Vitro Cytotoxicity. Int. J. Pharm..

[43] Louderbough J.M.V., Schroeder J.A. (2011). Understanding the Dual Nature of CD44 in Breast Cancer Progression. Mol. Cancer Res..

[44] Al-Othman N., Alhendi A., Ihbaisha M., Barahmeh M., Alqaraleh M., Al-Momany B.Z. (2020). Role of CD44 in Breast Cancer. Breast Dis..

[45] Dey A., Koli U., Dandekar P., Jain R. (2016). Investigating Behaviour of Polymers in Nanoparticles of Chitosan Oligosaccharides Coated with Hyaluronic Acid. Polymer.

[46] Park J.H., Cho H.J., Yoon H.Y., Yoon I.S., Ko S.H., Shim J.S., Cho J.H., Park J.H., Kim K., Kwon I.C. (2014). Hyaluronic Acid Derivative-Coated Nanohybrid Liposomes for Cancer Imaging and Drug Delivery. J. Control. Release.

[47] Gupta G., Asati P., Jain P., Mishra P., Mishra A., Singour P. (2022). Recent Advancements in Cancer Targeting Therapy with the Hyaluronic Acid as a Potential Adjuvant Avances Recientes En La Terapia Dirigida Al Cáncer Con El Ácido Hialurónico Como Adyuvante Potencial. Ars Pharm..

[48] Elamin K.M., Yamashita Y., Higashi T., Motoyama K., Arima H. (2018). Supramolecular Complex of Methyl-β-Cyclodextrin with Adamantane-Grafted Hyaluronic Acid as a Novel Antitumor Agent. Chem. Pharm. Bull..

[49] Elamin K.M., Motoyama K., Higashi T., Yamashita Y., Tokuda A., Arima H. (2018). Dual Targeting System by Supramolecular Complex of Folate-Conjugated Methyl-β-Cyclodextrin with Adamantane-Grafted Hyaluronic Acid for the Treatment of Colorectal Cancer. Int. J. Biol. Macromol..

[50] Bhattacharya D., Svechkrev D., Soucheck J., Hill T., Taylor M., Natarajan A., Mohs A. (2017). Impact of Structurally Modifying Hyaluronic Acid on CD44 Interaction. J. Mater. Chem. B.

[51] Jia Y., Chen S., Wang C., Sun T., Yang L. (2022). Hyaluronic Acid-Based Nano Drug Delivery Systems for Breast Cancer Treatment: Recent Advances. Front. Bioeng. Biotechnol..

[52] Nasti A., Zaki N.M., De Leonardis P., Ungphaiboon S., Sansongsak P., Rimoli M.G., Tirelli N. (2009). Chitosan/TPP and Chitosan/TPP-Hyaluronic Acid Nanoparticles: Systematic Optimisation of the Preparative Process and Preliminary Biological Evaluation. Pharm. Res..

[53] Almalik A., Donno R., Cadman C.J., Cellesi F., Day P.J., Tirelli N. (2013). Hyaluronic Acid-Coated Chitosan Nanoparticles: Molecular Weight-Dependent Effects on Morphology and Hyaluronic Acid Presentation. J. Control. Release.

[54] Nokhodi F., Nekoei M., Goodarzi M.T. (2022). Hyaluronic Acid-Coated Chitosan Nanoparticles as Targeted-Carrier of Tamoxifen against MCF7 and TMX-Resistant MCF7 Cells. J. Mater. Sci. Mater. Med..

[55] Aisha A.F.A., Abdulmajid A.M.S., Ismail Z., Alrokayan S.A., Abu-Salah K.M. (2015). Development of Polymeric Nanoparticles of Garcinia Mangostana Xanthones in Eudragit RL100/RS100 for Anti-Colon Cancer Drug Delivery. J. Nanomater..

[56] Smith B.C. (2011). Fundamentals of Fourier Transform Infrared Spectroscopy.

[57] Zak A., Majid W., ME A., Yousefi R. (2011). X-ray Analysis of ZnO Nanoparticles by Williamson–Hall and Size–Strain Plot Methods. Solid State Sci..

[58] CCRC U. (2012). Standard Procedure of the Cytotoxic Test MTT Method. Cancer Chemoprevention Res. Cent..

[59] Guo M., Wang X., Lu X., Wang H., Brodelius P.E. (2016). α-Mangostin Extraction from the Native Mangosteen (*Garcinia mangostana* L.) and the Binding Mechanisms of α-Mangostin to HSAorTRF. PLoS ONE.

[60] Rohman A., Arifah F.H., Irnawati, Alam G., Muchtaridi M. (2020). The Application of FTIR Spectroscopy and Chemometrics for Classification of Mangosteen Extract and Its Correlation with Alpha-Mangostin. J. Appl. Pharm. Sci..

[61] Zaman M., Butt M.H., Siddique W., Iqbal M.O., Nisar N., Mumtaz A., Nazeer H.Y., Alshammari A., Riaz M.S. (2022). Fabrication of PEGylated Chitosan Nanoparticles Containing Tenofovir Alafenamide: Synthesis and Characterization. Molecules.

[62] Oh J.W., Chun S.C., Chandrasekaran M. (2019). Preparation and in Vitro Characterization of Chitosan Nanoparticles and Their Broad-Spectrum Antifungal Action Compared to Antibacterial Activities against Phytopathogens of Tomato. Agronomy.

[63] Carneiro J., Döll-Boscardin P.M., Fiorin B.C., Nadal J.M., Farago P.V., De Paula J.P. (2016). Development and Characterization of Hyaluronic Acid-Lysine Nanoparticles with Potential as Innovative Dermal Filling. Braz. J. Pharm. Sci..

[64] Hussain Z., Pandey M., Choudhury H., Ying P.C., Xian T.M., Kaur T., Jia G.W., Gorain B. (2020). Hyaluronic Acid Functionalized Nanoparticles for Simultaneous Delivery of Curcumin and Resveratrol for Management of Chronic Diabetic Wounds: Fabrication, Characterization, Stability and in Vitro Release Kinetics. J. Drug Deliv. Sci. Technol..

[65] Iqbal A., Muhammad Shuib N.A., Darnis D.S., Miskam M., Abdul Rahman N.R., Adam F. (2018). Synthesis and Characterisation of Rice Husk Ash Silica Drug Carrier for α-Mangostin. J. Phys. Sci..

[66] Sriyanti I., Edikresnha D., Rahma A., Munir M.M., Rachmawati H., Khairurrijal K. (2018). Mangosteen Pericarp Extract Embedded in Electrospun PVP Nanofiber Mats: Physicochemical Properties and Release Mechanism of α-Mangostin. Int. J. Nanomed..

[67] Mulia K., Rachman D., Krisanti E.A. (2019). Preparation, Characterization and Release Profile of Chitosan Alginate Freeze Dried Matrices Loaded with Mangostins. Journal of Physics: Conference Series.

[68] Eddya M., Tbib B., EL-Hami K. (2020). A Comparison of Chitosan Properties after Extraction from Shrimp Shells by Diluted and Concentrated Acids. Heliyon.

[69] Podgorbunskikh E., Kuskov T., Rychkov D., Lomovskii O., Bychkov A. (2022). Mechanical Amorphization of Chitosan with Different Molecular Weights. Polymers.

[70] Jain A., Jain S.K., Ganesh N., Barve J., Beg A.M. (2010). Design and Development of Ligand-Appended Polysaccharidic Nanoparticles for the Delivery of Oxaliplatin in Colorectal Cancer. Nanomed. Nanotechnol. Biol. Med..

[71] Muhamad N., Plengsuriyakarn T., Na-Bangchang K. (2018). Application of Active Targeting Nanoparticle Delivery System for Chemotherapeutic Drugs and Traditional/Herbal Medicines in Cancer Therapy: A Systematic Review. Int. J. Nanomed..

[72] Campos J., Varas-Godoy M., Haidar Z.S. (2017). Physicochemical Characterization of Chitosan-Hyaluronan-Coated Solid Lipid Nanoparticles for the Targeted Delivery of Paclitaxel: A Proof-of-Concept Study in Breast Cancer Cells. Nanomedicine.

[73] Almeida P.V., Shahbazi M.A., Mäkilä E., Kaasalainen M., Salonen J., Hirvonen J., Santos H.A. (2014). Amine-Modified Hyaluronic Acid-Functionalized Porous Silicon Nanoparticles for Targeting Breast Cancer Tumors. Nanoscale.

[74] Almalik A., Karimi S., Ouasti S., Donno R., Wandrey C., Day P.J., Tirelli N. (2013). Hyaluronic Acid (HA) Presentation as a Tool to Modulate and Control the Receptor-Mediated Uptake of HA-Coated Nanoparticles. Biomaterials.

[75] Subhan M.A., Yalamarty S.S.K., Filipczak N., Parveen F., Torchilin V.P. (2021). Recent Advances in Tumor Targeting via Epr Effect for Cancer Treatment. J. Pers. Med..

[76] Chiesa E., Dorati R., Conti B., Modena T., Cova E., Meloni F., Genta I. (2018). Hyaluronic Acid-Decorated Chitosan Nanoparticles for CD44-Targeted Delivery of Everolimus. Int. J. Mol. Sci..

[77] Pereira F.M., Melo M.N., Santos Á.K.M., Oliveira K.V., Diz F.M., Ligabue R.A., Morrone F.B., Severino P., Fricks A.T. (2021). Hyaluronic Acid-Coated Chitosan Nanoparticles as Carrier for the Enzyme/Prodrug Complex Based on Horseradish Peroxidase/Indole-3-Acetic Acid: Characterization and Potential Therapeutic for Bladder Cancer Cells. Enzym. Microb. Technol..

[78] Samprasit W., Akkaramongkolporn P., Jaewjira S., Opanasopit P. (2018). Design of Alpha Mangostin-Loaded Chitosan/Alginate Controlled-Release Nanoparticles Using Genipin as Crosslinker. J. Drug Deliv. Sci. Technol..

[79] Polexe R.C., Delair T. (2013). Elaboration of Stable and Antibody Functionalized Positively Charged Colloids by Polyelectrolyte Complexation between Chitosan and Hyaluronic Acid. Molecules.

[80] Herdiana Y., Wathoni N., Shamsuddin S., Muchtaridi M. (2022). Drug Release Study of the Chitosan-Based Nanoparticles. Heliyon.

[81] Ofridam F., Tarhini M., Lebaz N., Gagnière E., Ofridam F., Tarhini M., Lebaz N., Gagnière E., Mangin D., Ofridam F. (2021). PH-Sensitive Polymers: Classification and Some Fine Potential Applications To Cite This Version: HAL Id: Hal-03132353 PH-Sensitive Polymers: Classification and Some Fine Potential Applications. Polym. Adv. Technol..

[82] Bai X., Smith Z.L., Wang Y., Butterworth S., Tirella A. (2022). Sustained Drug Release from Smart Nanoparticles in Cancer Therapy: A Comprehensive Review. Micromachines.

[83] Nunes D., Andrade S., Ramalho M.J., Loureiro J.A., Pereira M.C. (2022). Polymeric Nanoparticles-Loaded Hydrogels for Biomedical Applications: A Systematic Review on In Vivo Findings. Polymers.

[84] Azadi A., Hamidi M., Khoshayand M.R., Amini M., Rouini M.R. (2012). Preparation and Optimization of Surface-Treated Methotrexate-Loaded Nanogels Intended for Brain Delivery. Carbohydr. Polym..

[85] Kim S.W., Oh K.T., Youn Y.S., Lee E.S. (2014). Hyaluronated Nanoparticles with PH- and Enzyme-Responsive Drug Release Properties. Colloids Surfaces B Biointerfaces.

[86] Ullah F., Shah K.U., Shah S.U., Nawaz A., Nawaz T., Khan K.A., Alserihi R.F., Tayeb H.H., Tabrez S., Alfatama M. (2022). Synthesis, Characterization and In Vitro Evaluation of Chitosan Nanoparticles Physically Admixed with Lactose Microspheres for Pulmonary Delivery of Montelukast. Polymers.

[87] Saleh A., Akkuş-Dağdeviren Z.B., Friedl J.D., Knoll P., Bernkop-Schnürch A. (2022). Chitosan—Polyphosphate Nanoparticles for a Targeted Drug Release at the Absorption Membrane. Heliyon.

[88] Manimaran D., Elangovan N., Mani P., Subramanian K., Ali D., Alarifi S., Palanisamy C.P., Zhang H., Rangasamy K., Palanisamy V. (2022). Isolongifolene-Loaded Chitosan Nanoparticles Synthesis and Characterization for Cancer Treatment. Sci. Rep..

[89] Waqas M.K., Safdar S., Buabeid M., Ashames A., Akhtar M., Murtaza G. (2022). Alginate-Coated Chitosan Nanoparticles for PH-Dependent Release of Tamoxifen Citrate. J. Exp. Nanosci..

[90] Tapdiqov S., Taghiyev D., Zeynalov N., Safaraliyeva S., Fatullayeva S., Hummetov A., Raucci M., Mustafayev M., Jafarova R., Shirinova K. (2022). Cumulative Release Kinetics of Levothyroxine-Na Pentahydrate from Chitosan/Arabinogalactane Based PH Sensitive Hydrogel and It’s Toxicology. React. Funct. Polym..

[91] Herdiana Y., Wathoni N., Shamsuddin S., Muchtaridi M. (2022). Cytotoxicity Enhancement in MCF-7 Breast Cancer Cells with Depolymerized Chitosan Delivery of α-Mangostin. Polymers.

